# Safety of Early Discontinuation of Antiseizure Medication After Acute Symptomatic Neonatal Seizures

**DOI:** 10.1001/jamaneurol.2021.1437

**Published:** 2021-05-24

**Authors:** Hannah C. Glass, Janet S. Soul, Taeun Chang, Courtney J. Wusthoff, Catherine J. Chu, Shavonne L. Massey, Nicholas S. Abend, Monica Lemmon, Cameron Thomas, Adam L. Numis, Ronnie Guillet, Julie Sturza, Nancy A. McNamara, Elizabeth E. Rogers, Linda S. Franck, Charles E. McCulloch, Renée A. Shellhaas

**Affiliations:** 1 Department of Neurology and Weill Institute for Neuroscience, University of California, San Francisco; 2 Department of Pediatrics, UCSF Benioff Children’s Hospital, University of California, San Francisco; 3 Department of Epidemiology & Biostatistics; University of California, San Francisco; 4 Department of Neurology, Boston Children’s Hospital, Harvard Medical School, Boston, Massachusetts; 5 Department of Neurology, Children’s National Hospital, George Washington University School of Medicine, Washington, DC; 6 Department of Neurology, Stanford University, Palo Alto, California; 7 Division of Neonatal and Developmental Medicine, Department of Pediatrics, Stanford University, Palo Alto, California; 8 Department of Neurology, Massachusetts General Hospital, Harvard Medical School, Boston, Massachusetts; 9 Department of Neurology, Children’s Hospital of Philadelphia, Perelman School of Medicine at the University of Pennsylvania, Philadelphia; 10 Department of Pediatrics, Children’s Hospital of Philadelphia, Perelman School of Medicine at the University of Pennsylvania, Philadelphia; 11 Department of Anesthesia and Critical Care Medicine, Children’s Hospital of Philadelphia, Perelman School of Medicine at the University of Pennsylvania, Philadelphia; 12 Departments of Pediatrics, Duke University School of Medicine, Durham, North Carolina; 13 Department of Population Health Sciences, Duke University School of Medicine, Durham, North Carolina; 14 Department of Pediatrics, University of Cincinnati, Division of Neurology, Cincinnati Children’s Hospital Medical Center, Cincinnati, Ohio; 15 Division of Neonatology, Department of Pediatrics, Golisano Children's Hospital, University of Rochester, Rochester, New York; 16 Department of Pediatrics, University of Michigan, Ann Arbor; 17 Department of Family Health Care Nursing, University of California, San Francisco

## Abstract

**Importance:**

Antiseizure medication (ASM) treatment duration for acute symptomatic neonatal seizures is variable. A randomized clinical trial of phenobarbital compared with placebo after resolution of acute symptomatic seizures closed early owing to low enrollment.

**Objective:**

To assess whether ASM discontinuation after resolution of acute symptomatic neonatal seizures and before hospital discharge is associated with functional neurodevelopment or risk of epilepsy at age 24 months.

**Design, Setting, and Participants:**

This comparative effectiveness study included 303 neonates with acute symptomatic seizures (282 with follow-up data and 270 with the primary outcome measure) from 9 US Neonatal Seizure Registry centers, born from July 2015 to March 2018. The centers all had level IV neonatal intensive care units and comprehensive pediatric epilepsy programs. Data were analyzed from June 2020 to February 2021.

**Exposures:**

The primary exposure was duration of ASM treatment dichotomized as ASM discontinued vs ASM maintained at the time of discharge from the neonatal seizure admission. To enhance causal association, each outcome risk was adjusted for propensity to receive ASM at discharge. Propensity for ASM maintenance was defined by a logistic regression model including seizure cause, gestational age, therapeutic hypothermia, worst electroencephalogram background, days of electroencephalogram seizures, and discharge examination (all *P* ≤ .10 in a joint model except cause, which was included for face validity).

**Main Outcomes and Measures:**

Functional neurodevelopment was assessed by the Warner Initial Developmental Evaluation of Adaptive and Functional Skills (WIDEA-FS) at 24 months powered for propensity-adjusted noninferiority of early ASM discontinuation. Postneonatal epilepsy, a prespecified secondary outcome, was defined per International League Against Epilepsy criteria, determined by parent interview, and corroborated by medical records.

**Results:**

Most neonates (194 of 303 [64%]) had ASM maintained at the time of hospital discharge. Among 270 children evaluated at 24 months (mean [SD], 23.8 [0.7] months; 147 [54%] were male), the WIDEA-FS score was similar for the infants whose ASMs were discontinued (101 of 270 [37%]) compared with the infants with ASMs maintained (169 of 270 [63%]) at discharge (median score, 165 [interquartile range, 150-175] vs 161 [interquartile range, 129-174]; *P* = .09). The propensity-adjusted average difference was 4 points (90% CI, −3 to 11 points), which met the a priori noninferiority limit of −12 points. The epilepsy risk was similar (11% vs 14%; *P* = .49), with a propensity-adjusted odds ratio of 1.5 (95% CI, 0.7-3.4; *P* = .32).

**Conclusions and Relevance:**

In this comparative effectiveness study, no difference was found in functional neurodevelopment or epilepsy at age 24 months among children whose ASM was discontinued vs maintained at hospital discharge after resolution of acute symptomatic neonatal seizures. These results support discontinuation of ASM prior to hospital discharge for most infants with acute symptomatic neonatal seizures.

## Key Points

**Question:**

Is discontinuation of antiseizure medication (ASM) after resolution of acute symptomatic neonatal seizures and prior to discharge from the hospital associated with functional neurodevelopment or epilepsy at 24 months?

**Findings:**

In this comparative effectiveness study of 303 children with neonatal seizures from 9 centers, 64% had ASM maintained at hospital discharge. No difference was found between ASM maintenance and discontinuation groups in functional neurodevelopment or epilepsy; 13% of children developed epilepsy, including more than one-third with infantile spasms.

**Meaning:**

These results support discontinuing ASMs for most neonates with acute symptomatic seizures prior to discharge from the hospital, an approach that may represent an evidence-based change in practice for many clinicians.

## Teaser

This comparative effectiveness study assesses whether discontinuation of antiseizure medication prior to discharge from the hospital after resolution of acute symptomatic neonatal seizures is associated with impaired functional neurodevelopment or the risk of epilepsy at 24 months.

## Introduction

Neonatal seizures owing to brain injury (acute symptomatic seizures) are typically self-limited in the neonatal period; however, infants who survive neonatal seizures are at risk for postneonatal epilepsy (chronic unprovoked seizures).^1,2,3,4,5^ Although acute symptomatic neonatal seizures typically remit within 72 hours,^6^ infants often have antiseizure medications (ASMs) maintained for months to years because clinicians and parents may be concerned about the risks of continued seizures and early-life epilepsy.^7,8^ Thus, infants are often exposed to ASM (particularly phenobarbital, which is the most commonly used ASM for neonatal seizures^9,10,11^) for several months despite the lack of evidence that this exposure prevents epilepsy and despite data that phenobarbital has potential neurotoxic effects^12^ and may be associated with lower cognitive scores with long-term use.^13,14^

Results from small and single-center studies suggest that early ASM discontinuation for acute seizures is not harmful.^7,13,15,16,17^ However, this practice has not been widely adopted, including at Neonatal Seizure Registry (NSR) centers.^7,8^ Parents of infants affected by neonatal seizures identify ASM treatment duration as a high-priority research topic.^18^ In 2009, the National Institutes of Health funded a trial to randomize infants to receive phenobarbital or placebo for 4 months after acute neonatal seizure resolution (NCT01089504). The trial closed early because of reluctance from both parents and clinicians to randomize newborns. The decision to decline to enroll was evenly distributed among those concerned about placebo (discontinuing too early) and those worried about effects of prolonged phenobarbital exposure.

We aimed to determine whether discontinuation of ASM prior to discharge from the hospital after resolution of acute symptomatic neonatal seizures was associated with impaired functional neurodevelopment or the risk of epilepsy at 24 months. We hypothesized that, after adjusting for propensity to maintain ASM at discharge, infants whose ASM was discontinued would have no difference in functional neurodevelopment or risk of postneonatal epilepsy at age 24 months compared with those for whom ASM was maintained at discharge from the neonatal seizure admission.

## Methods

### Study Design

This was a prospective, observational, multicenter comparative effectiveness study of infants with acute symptomatic neonatal seizures born between July 2015 and March 2018 and enrolled at 9 NSR centers (prospectively registered at NCT02789176). A total of 150 of 305 infants (49%) were enrolled during the neonatal admission (inpatient), and 155 of 305 (51%) were recruited prior to age 24 months from the first NSR cohort^9^ or from outpatient clinics at a participating center (outpatient). Data were analyzed from June 2020 to February 2021. This study followed the Patient-Centered Outcomes Research Institute (PCORI)–specified guidelines for comparative effectiveness studies.

Each NSR center has a level IV neonatal intensive care unit and a level IV comprehensive pediatric epilepsy program. All enrolled infants underwent continuous conventional electroencephalogram (cEEG) monitoring according to the American Clinical Neurophysiology Society guidelines.^19^ No study-specific neonatal seizure treatment pathway was provided. To maintain the observational comparative effectiveness design, ASM selection, dosing, and treatment duration were at the discretion of the local health care professionals. A 2018 study showed a uniform approach to initial seizure treatment at NSR centers.^8^

Neonates were enrolled after parents provided informed and written consent. The local institutional review board for each center approved the study protocol. The study was informed by the NSR Parent Advisory Panel, which included key parent and community stakeholders.^20,21^

### Inclusion and Exclusion Criteria

Enrollment criteria were (1) neonate with cEEG-confirmed seizure at the study center or referring hospital or (2) neonate treated with ASM for clinical events suspected to be seizures if the clinical history, including event semiologic features, supported the diagnosis of seizures, and (3) the seizures had an acute symptomatic cause (ie, hypoxic-ischemic encephalopathy, ischemic stroke, intracranial hemorrhage, or other acute brain injury). Neonates with events that were determined not to be seizures based on history, semiologic features, or cEEG were not enrolled. Neonates with transient reversible seizure causes (eg, hyponatremia, hypocalcemia, or hypoglycemia without brain injury) or neonatal-onset epilepsy syndromes were excluded.

### Neonatal Measurements

The primary exposure was duration of ASM treatment dichotomized as ASM discontinued vs ASM maintained at the time of discharge from the neonatal seizure admission. Timing and dose of ASM administration were extracted from the medication administration record. Study center investigators established demographic and clinical data, as well as primary seizure cause, based on medical record review. When 2 or more seizure causes were present, only the primary cause was considered.

Seizure exposure was determined by study center investigators based on electrographic seizures^22^ on local cEEG, categorized as (1) status epilepticus (>30 minutes of seizures within any 1-hour epoch),^22^ (2) frequent recurrent seizures not fulfilling the definition of status epilepticus, (3) at least 7 isolated seizures, (4) less than 7 seizures, or (5) no EEG seizures at the study center.^9^ Seizure resolution was defined as 24 hours of cEEG without seizures per American Clinical Neurophysiology Society guideline recommendations.^19^ The most abnormal conventional EEG background was determined based on the EEG report, categorized as (1) normal (explicitly described as such in the report), (2) mild/moderately abnormal, or (3) severely abnormal (flat trace, severe discontinuity, or burst suppression).

### Outcome Measures

Neurodevelopmental outcomes were assessed by parent interview using a standardized telephone survey when the child was 12 months’, 18 months’, and 24 months’ corrected age. Parent interviews were corroborated by medical record review.

The primary outcome was functional neurodevelopment at age 24 months, measured with the Warner Initial Developmental Evaluation of Adaptive and Functional Skills (WIDEA-FS),^23^ a 50-item questionnaire designed to assess adaptive skills including mobility, communication, social cognition, and self-care. The WIDEA-FS has good concurrent validity with the Bayley Scales of Infant and Toddler Development, 3rd edition (Bayley-III).^24^ The WIDEA-FS was administered by research staff who were blinded to the child’s medical history and ASM treatment duration. A child was considered to have functional impairment when the WIDEA-FS total score was greater than 2 SDs below the mean for age.

Postneonatal epilepsy, a prespecified secondary outcome, was defined per International League Against Epilepsy criteria.^25^ As an exploratory outcome, motor function was assessed using a modified Gross Motor Function Classification System.^26^ Functional motor impairment was defined as a Gross Motor Function Classification System score greater than II at age 24 months.

### Missing Data

Multiple imputation was used to estimate the primary outcome after accounting for loss to follow-up. For ASM treatment duration after hospital discharge, single imputation was applied using medical record review for missing parent-reported values.

### Statistical Analysis

#### Power and Sample Size

We adopted a noninferiority design, setting the margin at 7% of the 24-month average WIDEA-FS score of 172 (12 points). This margin is a conservative and clinically significant threshold: developmental delay greater than 33% (or 57 points on the WIDEA-FS at 24 months) is needed before a child may access developmental services.^27^ Under these assumptions, we calculated that 116 children were required to have 0.8 power that the lower limit of a 2-sided 90% CI would be above the a priori noninferiority limit. The initial funding period allowed 12 months of follow-up, which necessitated a larger sample size owing to test characteristics. After funding was extended to allow 24-month follow-up and before participants were enrolled, we decided to enroll a cohort of 300 newborns. This cohort size allowed us to take into account loss to follow-up and propensity score adjustment analysis,^28^ as well as assess secondary outcomes associated with family well-being (reported separately^20,21^) and longer-term outcomes (ongoing).

#### Propensity Score Adjustment

Propensity adjustment was used to improve causal association and address confounding by indication in estimating the effect of discontinuing compared with maintaining ASM prior to hospital discharge by accounting for covariates that estimated treatment duration.^29,30^ Backward stepwise regression was used to build the initial propensity model (initial inclusion *P* ≤ .10; final model inclusion adjusted *P* ≤ .10). We also used a machine learning method, the least absolute shrinkage and selection operator (LASSO), to select the variables, which gave propensity scores that were virtually identical to backward stepwise regression.^31^ The final model included gestational age, worst EEG background during the first 24 hours of recording, number of calendar days with EEG seizures, treatment with therapeutic hypothermia, and discharge neurological examination. Seizure cause was added to the final model for face validity.

The propensity model area under the curve was 0.74 (acceptable fit) and improved to 0.92 (outstanding fit) when study center was added. The Hosmer-Lemeshow Goodness-of-Fit Test indicated excellent fit. These results help confirm that we adequately accounted for possible confounders.^28,32^ Study center was not included in the propensity adjustment for the primary analyses because treatment variability by study center was the basis for comparison between otherwise similar clinical scenarios.

For the primary outcome (24-month WIDEA-FS score), we conducted a linear mixed-model analysis with random effects for intercepts and time using restricted maximum likelihood fitting and Kenward-Roger degree of freedom adjustments. The sole estimators in the regression models were ASM at discharge and propensity categorized as quintiles. We calculated 90% CIs with noninferiority established if the adjusted CI lay within the noninferiority range defined above. For the secondary outcome (epilepsy), we calculated odds ratios for postneonatal epilepsy before age 24 months and hazard ratios for time to epilepsy diagnosis with 95% CIs adjusted for propensity categorized as quintiles.

#### Sensitivity Analyses

As sensitivity analyses, we examined the center as an instrumental variable analysis (by fitting a random-effects instrumental variables panel model on each of the outcomes and using bootstrap standard errors to accommodate the use of a linear model for discharge on ASM within the instrumental variables routine) and interaction by recruitment group (inpatient vs outpatient).

A χ^2^ or Fisher exact test was used to test for the difference between categorical variables. Analysis of variance was used to test the difference between continuous variables for normally distributed data and a Kruskal-Wallis was used to determine the difference between continuous variables in nonnormally distributed data. Analyses were conducted in Stata, version 14 (StataCorp LLC) and SAS, version 9.4 (SAS Institute Inc). All tests were set at a significance level of *P* < .05. Noninferiority testing was 1-tailed and all others were 2-tailed.

## Results

**Figure 1.**
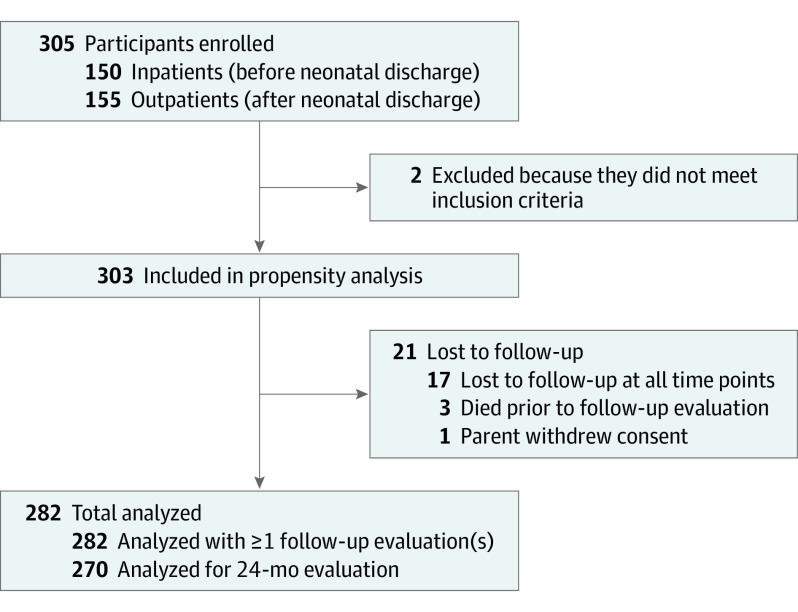
Flow Diagram of Study Participants With Neonatal Onset Acute Symptomatic Seizures

We enrolled 305 infants; 2 were later excluded (170 of 303 [56%] were male) (Figure 1). Seizure cause was hypoxic-ischemic encephalopathy in 130 (43%), ischemic stroke in 79 (26%), intracranial hemorrhage in 55 (18%), or other acute brain injury in 39 (13%), which included intracranial infection in 24, hypoglycemia with brain injury in 4, and uncategorized in 11. Phenobarbital was the first loading ASM for 90% (Table 1).

**Table 1.** Characteristics Considered for Propensity Adjustment for 303 Infants With Acute Symptomatic Neonatal Seizures and ASMs Discontinued or Maintained at Hospital Discharge

Characteristic	No. (%)			*P* value
	Total (N = 303)	ASM		
		Discontinued (n = 109)	Maintained (n = 194)	
**Clinical**				
Gestational age at birth, wk^a^				
<28	9 (3)	5 (5)	4 (2)	.10
28 to <32	6 (2)	3 (3)	3 (2)	
32 to <37	35 (12)	7 (6)	28 (14)	
≥37	253 (84)	94 (86)	159 (82)	
Male sex	170 (56)	59 (54)	111 (57)	.50
5-min Apgar score, median (IQR)	8 (5-9)	6 (4-9)	8 (6-9)	.002
Infant location at the time of seizure evaluation				
NICU	269 (89)	103 (94)	166 (86)	.70
PICU	9 (3)	3 (3)	6 (3)	
CICU	24 (8)	3 (3)	21 (11)	
Other	1 (<1)	0	1 (1)	
**Seizure and EEG**				
Seizure cause^a^				
Hypoxic-ischemic encephalopathy	130 (43)	58 (54)	72 (37)	.04
Ischemic stroke	79 (26)	22 (20)	57 (29)	
Intracranial hemorrhage	55 (18)	17 (16)	38 (20)	
Other	39 (13)	12 (11)	27 (14)	
Worst EEG background (first 24 h at study center)^a^				
Normal	25 (8)	14 (13)	11 (6)	.06
Mild/moderately abnormal	199 (66)	75 (69)	124 (64)	
Severely abnormal (burst suppression, depressed/undifferentiated, flat tracing)	53 (17)	14 (13)	39 (20)	
Status epilepticus at onset of recording	24 (8)	6 (6)	18 (9)	
Cannot assess	2 (<1)	0	2 (1)	
EEG seizure frequency (at the study center)				
None	52 (17)	26 (24)	26 (13)	.02
Few (<7)	83 (27)	35 (32)	48 (25)	
Many isolated (≥7)	58 (19)	20 (18)	38 (19)	
Frequent recurrent	64 (21)	15 (14)	49 (25)	
Status epilepticus	45 (15)	13 (12)	32 (16)	
Documentation inadequate	1 (<1)	0	1 (1)	
Days of EEG seizures, median (IQR)^a^	1 (1-2)	1 (1-2)	2 (1-2)	<.001
Initial loading ASM				
Phenobarbital	273 (90)	96 (88)	177 (91)	.007
Levetiracetam	17 (6)	3 (3)	14 (7)	
Fosphenytoin	3 (1)	2 (2)	1 (1)	
No loading dose	10 (3)	8 (7)	2 (1)	
Incomplete response to initial loading dose of ASM	186 (62)	58 (54)	128 (66)	.06
Received ≥2 ASMs to treat neonatal seizures	160 (53)	49 (45)	111 (57)	.04
Total inpatient PB exposure, median (IQR), mg/kg	63 (45-105)	48 (29-61)	76 (54-126)	<.001
Clinical course				
Complex medical diagnosis (congenital heart disease, ECMO, congenital diaphragmatic hernia)	36 (12)	8 (7)	28 (14)	.07
Therapeutic hypothermia^a^	86 (28)	44 (40)	43 (22)	.001
Abnormal neurologic examination results at discharge^a^	94 (31)	20 (18)	74 (38)	<.001

Abbreviations: ASM, antiseizure medication; CICU cardiac intensive care unit; ECMO extracorporeal membrane oxygenation; EEG, electroencephalogram; IQR, interquartile range; NICU, neonatal intensive care unit; PB, phenobarbital; PICU pediatric intensive care unit.

^a^ Included in the final propensity model.

### ASM at Discharge

At the time of discharge from the neonatal seizure admission, ASMs were maintained for 64% of neonates (194 of 303; range across study centers, 10%-95%; *P* < .001). Among these neonates, 131 of 194 (68%) had phenobarbital monotherapy maintained, 25 of 194 (13%) had levetiracetam monotherapy maintained, and 38 of 194 (20%) had polytherapy maintained.

Infants with high seizure burden, complex clinical course, and abnormal findings on the discharge neurological examination had a higher propensity for ASM maintenance at the time of hospital discharge (Table 1); these variables were incorporated into the propensity score. There was good overlap agreement in clinical characteristics within propensity quintiles and in propensity scores between the ASM maintenance groups (eTable 1 and eFigure in the Supplement). Among neonates whose ASMs were discontinued before hospital discharge, median treatment duration was 6 days (interquartile range [IQR], 3-11 days). Among children whose ASMs were maintained, duration of therapy was a median of 4 months (IQR, 3-8 months; *P* < .001). For the subset of full-term neonates with seizures owing to hypoxic-ischemic encephalopathy (excluding 46 infants with congenital heart malformations), the results were similar (ASM duration, 6 days [IQR, 3-9 days] when discontinued before hospital discharge vs 4 months [IQR, 3-5 months] when maintained at discharge; *P* < .001).

Three infants died after hospital discharge and 1 child’s parent withdrew the child from the study, leaving 299 eligible for follow-up. Among these children, 282 of 299 (94%) had follow-up at 1 or more points (Figure 1). Key clinical characteristics were not associated with loss to follow-up (eTable 2 in the Supplement).

### Functional Neurodevelopment

#### Unadjusted Analysis

**Figure 2.**
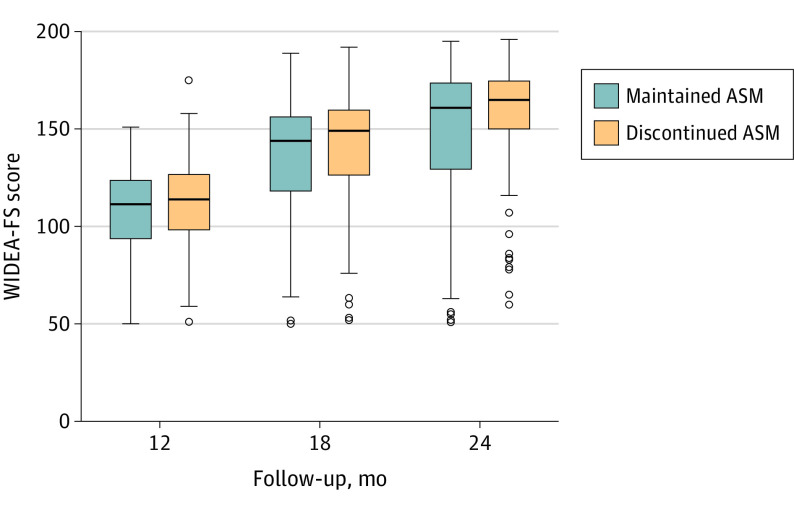
Unadjusted Functional Neurodevelopment Among 282 Infants With Acute Symptomatic Neonatal Seizures Unadjusted Warner Initial Developmental Evaluation of Adaptive and Functional Skills (WIDEA-FS) scores at 12 months’, 18 months’, and 24 months’ corrected age among 282 infants with acute symptomatic neonatal seizures whose antiseizure medications (ASMs) were discontinued (orange) vs maintained (blue) at the time of discharge from the neonatal seizure admission. The mean (SD) WIDEA-FS score in typically developing children is 109 (17) at 12 months, 152 (16) at 18 months, and 172 (10) at 24 months.

Among 270 children evaluated at 24 months (mean [SD], 23.8 [0.7] months; 147 [54%] were male), the median 24-month WIDEA-FS score was 164 (IQR, 136-175). Unadjusted total WIDEA-FS scores were 4 points (2%) higher for children whose ASM was discontinued prior to hospital discharge (101 of 270 [37%]) compared with children whose ASM was maintained at discharge (169 of 270 [63%]) (median score, 165 [IQR, 150-175] vs the median score, 161 [IQR, 129-174]; *P* = .09) (Table 2). The proportion of children with impaired functional neurodevelopment at 24 months was not different between the treatment duration groups (28% vs 37%; odds ratio, 0.6; 95% CI, 0.4-1.1; *P* = .11). The mean (SD) WIDEA-FS score in typically developing children is 109 (17) at 12 months, 152 (16) at 18 months, and 172 (10) at 24 months (Figure 2).

**Table 2.** Unadjusted and Propensity-Adjusted 2-Year Outcomes for 282 Neonates With Acute Symptomatic Seizures

Variable	ASM, median (IQR)		Unadjusted (95% CI)	Unadjusted *P* value	Adjusted (90% CI)	Adjusted *P* value
	Discontinued (n = 106)	Maintained (n = 176)				
WIDEA-FS						
12 mo^a^	114 (98 to 127)	112 (94 to 124)	Difference, 5 (−1 to 11)	.13	Difference, 1 (−4 to 7)	.70
18 mo^b^	149 (126 to 160)	144 (118 to 157)	Difference, 7 (0 to 14)	.04	Difference, 4 (−2 to 10)	.31
24 mo^c^	165 (150 to 175)	161 (129 to 174)	Difference, 7 (−1 to 15)	.09	Difference, 4 (−3 to 11)	.40
Postneonatal epilepsy, No. (%)	12 (11)	25 (14)	OR, 0.8 (0.4 to 1.6)	.49	OR, 1.5 (0.7 to 3.4)	.32
Motor impairment (GMFCS ≥II), No. (%)	13 (13)	32 (19)	OR, 0.6 (0.3 to 1.3)	.18	OR, 0.9 (0.4 to 1.9)	.71

Abbreviations: ASM, antiseizure medication; GMFCS, Gross Motor Function Classification System; IQR, interquartile range; OR, odds ratio; WIDEA-FS, Warner Initial Developmental Evaluation of Adaptive and Functional Skills.

^a^ n = 187 at 12 months.

^b^ n = 220 at 18 months.

^c^ n = 270 at 24 months.

#### Propensity-Adjusted Analysis

The propensity-adjusted difference in WIDEA-FS scores at age 24 months (primary outcome) remained 4 points (2%) higher among infants whose ASM was discontinued vs maintained at the time of hospital discharge (90% CI, −3 to 11), which met our a priori noninferiority limit of −12 points (Table 2). Multiple imputation analysis including children lost to follow-up did not change this result.

#### Prespecified Subgroup Analyses

Among the 50 infants born prematurely (<37 weeks of gestational age), the propensity-adjusted estimated WIDEA-FS score difference was 14 points higher among those whose ASM was discontinued vs maintained at hospital discharge (90% CI, −11 to 39). Among the 130 infants with hypoxic-ischemic encephalopathy as the neonatal seizure cause, the propensity-adjusted estimated WIDEA-FS score difference was 10 points higher among those whose ASM was discontinued vs maintained at hospital discharge (90% CI, 0-20).

#### Sensitivity Analyses

Using center as an instrumental variable and fitting a model comparable to the propensity-adjusted mixed model produced virtually identical results (estimated adjusted WIDEA-FS score, 3 points higher [95% CI, −1 to 8] among those whose ASMs were discontinued vs maintained at hospital discharge). Tests of interaction showed no difference in outcomes for the infants recruited as inpatients vs outpatients (estimated adjusted WIDEA-FS score in a model including the interaction term, 9 points higher [95% CI, −1 to 19] among those whose ASMs were discontinued vs maintained at hospital discharge).

### Epilepsy

#### Unadjusted Analysis

Thirteen percent of infants (37 of 282) developed epilepsy before age 24 months, including 5% with infantile spasms (13 of 282). The median age at epilepsy onset was 7 months (IQR, 3-14 months). No infant with a normal EEG background developed epilepsy (0 of 23), and 13 of 195 (7%) of those with a normal neurological examination at discharge developed epilepsy. None of the children classified as low risk according to the World Health Organization (0 of 17 with both normal EEG results and normal neurological examination at discharge) developed epilepsy compared with 14% of children (37 of 265) who were not low risk (*P* = .10).

**Figure 3.**
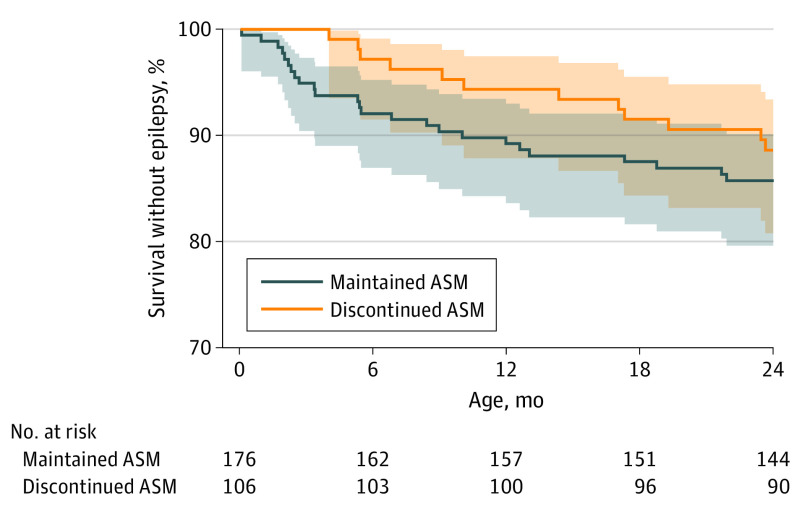
Unadjusted Epilepsy-Free Survival Among 282 Infants With Acute Symptomatic Neonatal Seizures No difference in epilepsy-free survival among infants with acute symptomatic neonatal seizures for whom antiseizure medications (ASMs) were discontinued (orange) vs maintained (blue) at the time of discharge (hazard ratio, 0.8; 95% CI, 0.4-1.5).

The risk of epilepsy did not differ by ASM treatment duration group (11% for infants whose ASM was discontinued vs 14% for those whose ASM was maintained at the time of hospital discharge: odds ratio, 0.8; 95% CI, 0.4-1.6; *P* = .49) (Table 2) or timing of epilepsy onset (hazard ratio, 0.8; 95% CI, 0.4-1.5) (Figure 3**)**. All 11 children with epilepsy onset before 4 months had ASM maintained at hospital discharge, and 4 of 11 (36%) had infantile spasms.

#### Propensity-Adjusted Analysis

After propensity adjustment, there was no difference in the risk of epilepsy (adjusted odds ratio, 1.5; 95% CI, 0.7-3.4; *P* = .32) (Table 2) or age at epilepsy onset (adjusted hazard ratio, 1.4; 95% CI, 0.7-2.9; *P* = .37) for infants whose ASM was discontinued vs maintained at hospital discharge.

### Motor Function

#### Unadjusted Analysis

Overall, 16% of infants (43 of 270) had a Gross Motor Function Classification System score greater than or equal to II, with no difference between infants whose ASM was discontinued prior to vs maintained at hospital discharge (13% vs 19%; odds ratio, 0.6; 95% CI, 0.3-1.3; *P* = .18) (Table 2).

#### Propensity-Adjusted Analysis

After propensity adjustment, the risk of a Gross Motor Function Classification System score greater than or equal to II did not significantly differ by ASM treatment duration (adjusted odds ratio, 0.9; 95% CI, 0.4-1.9; *P* = .71) (Table 2).

## Discussion

In this prospective observational multicenter comparative effectiveness study of neonates with acute symptomatic seizures, discontinuation of ASM after resolution of acute seizures and before hospital discharge was safe. The ASM discontinuation had no adverse effect on the primary outcome, functional neurodevelopment at 24 months, when adjusted for ASM treatment duration propensity as determined a priori by a no more than 7% difference on the WIDEA-FS. The WIDEA-FS score was chosen for its ease of telephone administration and concurrent validity with Bayley-III.^24^ Similarly, there was no significant association between ASM treatment duration and development of postneonatal epilepsy or gross motor function at age 24 months. These results are not associated with differences in neonatal clinical characteristics. The propensity analysis enhanced causal association and adjusted for key clinical covariates, such as seizure severity and neurological examination, factors that may affect clinician choice regarding ASM treatment duration. The results, therefore, support discontinuation of ASMs in most neonates with acute symptomatic seizures prior to discharge from the hospital. The World Health Organization recommends that clinicians consider discontinuing ASMs without a taper after 72 hours of seizure freedom for neonates with a normal neurological examination and EEG.^33^ We expand this recommendation to include all neonates with acute symptomatic seizures, even in the setting of abnormal EEG and neurological examination. Adopting this approach may represent an evidence-based change in practice for many clinicians.

There is a latent (seizure-free) period between the resolution of acute symptomatic neonatal seizures and emergence of postneonatal epilepsy. With a follow-up period of 24 months, our data showed that the median age at epilepsy onset was 7 months. Notably, none of the children for whom ASM was discontinued had recurrent seizures in the weeks after discharge. All 11 children with early-onset unprovoked seizures (epilepsy before 4 months) were in the group for whom ASM was maintained at discharge. Furthermore, one-third of children with epilepsy onset before 4 months developed infantile spasms, a seizure type for which phenobarbital and levetiracetam are ineffective.^34^ Our results support conclusions from smaller single-center studies that ASMs used to treat neonatal seizures do not appear to affect the duration of the latent period or overall risk of postneonatal epilepsy.^15,17,35^ These data raise questions about the rationale for long-term use of ASM after resolution of acute symptomatic neonatal seizures.

The practice of continuing ASM for infants with neonatal seizures was established prior to recommended standard use of diagnostic EEG and magnetic resonance imaging for all neonates with suspected seizures.^36^ Contemporary neurocritical care management uses cEEG to accurately diagnose seizures and ASM response^19,37,38^ along with magnetic resonance imaging and genetic testing to determine the cause of seizures, which allows for a tailored management approach. Neonates with paroxysmal events that are not seizures do not need ASMs; some of these children may have been included in older studies.^38^ Conversely, children with neonatal-onset epilepsy are likely to need long-term ASMs and may benefit from precision treatments.^39,40,41,42^ In addition, neonates with complex clinical course and prolonged hospitalization may rarely evolve from acute symptomatic seizures to unprovoked seizures (epilepsy) prior to their first hospital discharge; these infants may also require ongoing daily ASM treatment.

Our results are consistent with a prior study showing that discontinuation of ASMs prior to hospital discharge results in decreased exposure to phenobarbital,^43^ a drug that may be harmful with prolonged use.^13^ Among neonates in this study whose ASMs were discontinued prior to discharge, the total inpatient exposure to phenobarbital was, on average, 28 mg/kg less than for infants for whom it was maintained at the time of hospital discharge. After accounting for medication taken after discharge, infants with ASMs discontinued prior to discharge received 94% fewer days of phenobarbital treatment than those whose ASMs were maintained.

This study was not powered to determine whether long-term treatment with levetiracetam is preferable to continued phenobarbital. A small subset of infants received levetiracetam as part of their ASM regimen, almost always in combination with phenobarbital (a minority were discharged while receiving levetiracetam monotherapy). Data from the NEOLEV2 trial^44^ suggest that levetiracetam is far less effective than phenobarbital for initial neonatal seizure control; our data add that it is safe to discontinue phenobarbital when used for first-line treatment; therefore, there is no need to switch a child to a less sedating medication, such as levetiracetam, prior to discharge.

### Limitations

This study has limitations. First, while we were well powered to determine noninferiority for our primary outcome of 24-month functional neurodevelopment, postneonatal epilepsy was a rare outcome (13%). Although there was no significant difference in epilepsy among the treatment duration groups, the cohort size does not allow us to exclude a difference of up to 3.4 times the odds of developing epilepsy before age 24 months. Larger longer-term studies are needed. Second, follow-up was limited to 24 months. While some children may develop epilepsy after 24 months, we speculate that neonatal ASM duration would not modulate risk of childhood-onset epilepsy. Third, 17% of included neonates did not have EEG seizures at the study center, although many had EEG-confirmed seizures at referral centers. We included these children for generalizability because even children who never have EEG seizures may receive ASM long after hospital discharge. Experts agree that any study evaluating the efficacy of an ASM should include only children with EEG seizures^45^; however, this study did not evaluate the efficacy of acute seizure treatment but rather the safety of its discontinuation.

## Conclusions

Results of this comparative effectiveness study suggest that the discontinuation of ASM after resolution of acute symptomatic neonatal seizures and prior to discharge from the seizure admission is safe: at age 24 months, functional neurodevelopmental outcome and the rate of epilepsy were similar between the treatment duration groups. Our data suggest that prolonged ASM treatment is unnecessary for most neonates and support routine discontinuation of ASMs after resolution of acute symptomatic neonatal seizures prior to hospital discharge, which may require a change in practice at most centers.

## Notes

Supplement. **eTable 1.** Balance of Participant Characteristic Between Groups Within Quintiles of the Propensity Score for 303 Children Whose Antiseizure Medications Were Discontinued vs Maintained After Resolution of Acute Symptomatic Neonatal Seizures and Before Hospital Discharge **eTable 2.** Characteristics of Children Lost to Follow-up as Compared to Survivors of Acute Symptomatic Neonatal Seizures With One or More Follow-up Evaluations **eFigure.** Overlaid Density Plots of Propensity Scores by Quintile for 303 Infants Whose Antiseizure Medications Were Discontinued vs Maintained Upon Discharge From the Neonatal Seizure Admission
