# Supplementary material for: Safety of Early Discontinuation of Antiseizure Medication After Acute Symptomatic Neonatal Seizures
Source: JAMA Neurol. 2021 May 24;78(7):1–9. doi: 10.1001/jamaneurol.2021.1437 (PMC8145161; doi:10.1001/jamaneurol.2021.1437)
Supplement: Supplement. — eTable 1. Balance of Participant Characteristic Between Groups Within Quintiles of the Propensity Score for 303 Children Whose Antiseizure Medications Were Discontinued vs Maintained After Resolution of Acute Symptomatic Neonatal Seizures and Before Hospital Discharge eTable 2. Characteristics of Children Lost to Follow-up as Compared to Survivors of Acute Symptomatic Neonatal Seizures With One or More Follow-up Evaluations eFigure. Overlaid Density Plots of Propensity Scores by Quintile for 303 Infants Whose Antiseizure Medications Were Discontinued vs Maintained Upon Discharge From the Neonatal Seizure Admission [file jamaneurol-e211437-s001.pdf]

Supplemental Online Content

Glass HC, Soul JS, Chang T, et al. Safety of early discontinuation of antiseizure medication after acute symptomatic neonatal seizures. *JAMA Neurol*. Published online May 24, 2021. doi:10.1001/jamaneurol.2021.1437

eTable 1. Balance of Participant Characteristic Between Groups Within Quintiles of the Propensity Score for 303 Children Whose Antiseizure Medications Were Discontinued vs Maintained After Resolution of Acute Symptomatic Neonatal Seizures and Before Hospital Discharge
eTable 2. Characteristics of Children Lost to Follow-up as Compared to Survivors of Acute Symptomatic Neonatal Seizures With One or More Follow-up Evaluations
eFigure. Overlaid Density Plots of Propensity Scores by Quintile for 303 Infants Whose Antiseizure Medications Were Discontinued vs Maintained Upon Discharge From the Neonatal Seizure Admission

This supplemental material has been provided by the authors to give readers additional information about their work.

© 2021 Glass HC et al. *JAMA Neurology*.

eTable 1: Balance of participant characteristic between groups within quintiles of the propensity score for 303 children whose antiseizure medications were discontinued vs. maintained after resolution of acute symptomatic neonatal seizures and before hospital discharge.

Propensity Score Quintiles	Quintile 1		Quintile 2		Quintile 3		Quintile 4		Quintile 5	
	ASM Discontinued N=43	ASM Maintained N=17	ASM Discontinued N=25	ASM Maintained N= 37	ASM Discontinued N=16	ASM Maintained N= 42	ASM Discontinued N=18	ASM Maintained N= 44	ASM Discontinued N=8	ASM Maintained N= 53
Clinical Characteristics										
Gestational age at birth[a], No. (%)										
- <28 weeks	4 (9%)	2 (12%)	1 (4%)	0 (0%)	0 (0%)	1 (2%)	0 (0%)	1 (2%)	0 (0%)	0 (0%)
-28- <32 weeks	3 (7%)	0 (0%)	0 (0%)	1 (3%)	0 (0%)	0 (0%)	0 (0%)	1 (2%)	0 (0%)	0 (0%)
-32- <37 weeks	1(2%)	1 (6%)	1 (4%)	3 (8%)	1 (6%)	0 (0%)	1 (6%)	7 (16%)	3 (38%)	17 (32%)
-≥37 weeks	35 (81%)	14 (82%)	23 (92%)	33 (89%)	15 (94%)	41 (98%)	17 (94%)	35 (80%)	5 (62%)	36 (68%)
Male, No. (%)	19 (44%)	8 (47%)	14 (56%)	21 (57%)	12 (75%)	23 (55%)	9 (50%)	30 (68%)	5 (62%)	29 (55%)
5-minute Apgar score, median (IQR)	5 (3,6)	5 (3,6)	8 (5,9)	7 (3,9)	9 (7,9)	9 (7,9)	6 (5,9)	9 (8,9)	7 (5,9)	8 (6,9)
Seizure and EEG Characteristics										
Seizure etiology[a], No. (%)										
-HIE	30 (70%)	14 (82%)	13 (52%)	22 (59%)	4 (25%)	10 (24%)	7 (39%)	11 (25%)	4 (50%)	15 (28%)
-Ischemic stroke	4 (9%)	1 (6%)	3 (12%)	3 (8%)	6 (38%)	22 (52%)	9 (50%)	15 (34%)	1 (12%)	16 (30%)
-ICH	4 (9%)	2 (12%)	4 (16%)	5 (14%)	6 (38%)	6 (14%)	2 (11%)	14 (32%)	1 (12%)	11 (21%)
-Other	5 (12%)	0 (0%)	5 (20%)	7 (19%)	0 (0%)	4 (10%)	0 (0%)	4 (9%)	2 (25%)	11 (21%)
Worst EEG background (1st 24hrs at study center)[a], No. (%)										
-Normal	11 (26%)	2 (12%)	3 (12%)	4 (11%)	0 (0%)	3 (7%)	0 (0%)	1 (2%)	0 (0%)	1 (2%)
-Mild/moderately abnormal	29 (67%)	11 (65%)	20 (80%)	26 (70%)	14 (89%)	31 (74%)	10 (56%)	29 (66%)	3 (38%)	27 (51%)
-Severely abnormal	2 (5%)	3 (18%)	2 (8%)	6 (16%)	1 (6%)	5 (12%)	6 (33%)	8 (18%)	3 (38%)	16 (30%)
-Status epilepticus	1 (2%)	1 (6%)	0 (0%)	1 (3%)	1 (6%)	3 (7%)	2 (11%)	6 (14%)	2 (25%)	7 (13%)
-Cannot assess	0 (0%)	0 (0%)	0 (0%)	0 (0%)	0 (0%)	0 (0%)	0 (0%)	0 (0%)	0 (0%)	2 (4%)
EEG seizure frequency (at the study center), No. (%)										
-None	16 (37%)	3 (18%)	6 (24%)	4 (11%)	3 (19%)	12 (29%)	0 (0%)	3 (7%)	1 (12%)	4 (8%)
-Few (<7)	17 (40%)	7 (41%)	9 (36%)	16 (43%)	2 (12%)	11 (26%)	5 (28%)	8 (18%)	2 (25%)	6(11%)
-Many isolated (≥7)	8 (19%)	5 (29%)	6 (24%)	7 (19%)	4 (25%)	10 (24%)	3 (17%)	6 (14%)	0 (0%)	9 (17%)
-Frequent recurrent	0 (0%)	0 (0%)	3 (12%)	8 (22%)	5 (31%)	5 (12%)	6 (33%)	19 (43%)	1 (12%)	17 (32%)
-Status Epilepticus	2 (5%)	2 (12%)	1 (4%)	1 (3%)	2 (12%)	4 (10%)	4 (22%)	8 (18%)	4 (50%)	17 (32%)
-Documentation inadequate	0 (0%)	0 (0%)	0 (0%)	1 (3%)	0 (0%)	0 (0%)	0 (0%)	0 (0%)	0 (0%)	0 (0%)
Days of EEG seizures[a], median (IQR)	1 (0,1)	1 (1,2)	1 (1,1)	1 (1,2)	1 (1,2)	1 (0,2)	2 (2,2)	2 (1,3)	1 (1,2)	2 (1,3)

© 2021 Glass HC et al. *JAMA Neurology*.

Incomplete response to initial loading dose of medication, No. (%)	14 (33%)	6 (35%)	13 (52%)	22 (59%)	12 (75%)	26 (62%)	14 (78%)	33 (75%)	6 (75%)	41 (77%)
Clinical course										
Therapeutic hypothermia[a], No. (%)	31 (72%)	14 (82%)	3 (12%)	13 (35%)	3 (19%)	6 (14%)	8 (44%)	5 (11%)	0 (0%)	4 (7%)
Abnormal neurologic exam at discharge[a], No. (%)	2 (5%)	0 (0%)	2 (8%)	6 (16%)	3 (19%)	12 (29%)	7 (38%)	13 (30%)	6 (75%)	44 (83%)

[a] indicates variables that were included in the propensity score.
ASM antiseizure medication; **HIE** hypoxic-ischemic encephalopathy; **ICH** intracranial hemorrhage; **EEG** electroencephalogram

© 2021 Glass HC et al. *JAMA Neurology*.

eTable 2. Characteristics of children lost to follow-up as compared to survivors of acute symptomatic neonatal seizures with one or more follow-up evaluations.

	Total N=299	Lost to f/u 17 (6%)	+f/u evaluation 282 (94%)	p-value
Clinical Characteristics				
Term, No. (%)	251 (84%)	13 (76%)	238 (84%)	0.3
Male, No. (%)	168 (56%)	12 (71%)	156 (55%)	0.2
5-minute Apgar score, median (IQR)	8 (5, 9)	8 (6, 9)	8 (5,9)	0.9
NICU at the time of seizure evaluation, No. (%)	267 (89%)	15 (88%)	252 (89%)	0.9
Seizure and EEG Characteristics				
Seizure etiology, No. (%)				
-Hypoxic-ischemic encephalopathy	129 (43%)	6 (35%)	123 (44%)	0.7
-Ischemic stroke	78 (26%)	2 (12%)	76 (27%)	
-Intracranial hemorrhage	54 (18%)	7 (41%)	47 (17%)	
-Other	38 (13%)	2 (12%)	36 (13%)	
Worst EEG background (1st 24hrs at study center), No. (%)				
-Normal	25 (8%)	2 (12%)	23 (8%)	0.5
-Mild/moderately abnormal	197 (66%)	10 (59%)	187 (66%)	
-Severely abnormal (burst suppression, depressed/undifferentiated, flat tracing)	51 (17%)	5 (29%)	46 (16%)	
-Status epilepticus at onset of recording	24 (8%)	0	24 (9%)	
-Cannot assess	2 (<1%)	0	2 (<1%)	
High seizure burden (≥7 EEG seizures at the study center), No. (%)	166 (56%)	10 (59%)	156 (55%)	0.8
Days of EEG seizures, median (IQR)	1 (1,2)	1(1,3)	2 (1,2)	0.4
Phenobarbital initial loading ASM, No. (%)	269 (90%)	15 (88%)	254 (90%)	0.6
Incomplete response to initial loading dose of medication, No. (%)	184 (62%)	10 (58%)	174 (62%)	0.8
Received ≥2 ASM to treat neonatal seizures, No. (%)	159 (53%)	9 (52%)	150 (53%)	0.98
Clinical course				
Complex medical diagnosis (congenital heart disease, ECMO, congenital diaphragmatic hernia), No. (%)	34 (11%)	1 (6%)	33 (12%)	0.5
Therapeutic hypothermia[a], No. (%)	86 (29%)	2 (12%)	84 (30%)	0.1
Abnormal neurologic exam at discharge[a], No. (%)	92 (31%)	5 (29%)	87 (31%)	0.9

ASM antiseizure medication; **NICU** neonatal intensive care unit; **PICU** pediatric intensive care unit; **CICU** cardiac intensive care unit; **EEG** electroencephalogram; **ECMO** extracorporeal membrane oxygenation

© 2021 Glass HC et al. *JAMA Neurology*.

eFigure. Overlaid density plots of propensity scores by quintile for 303 infants whose antiseizure medications were discontinued vs. maintained upon discharge from the neonatal seizure admission.

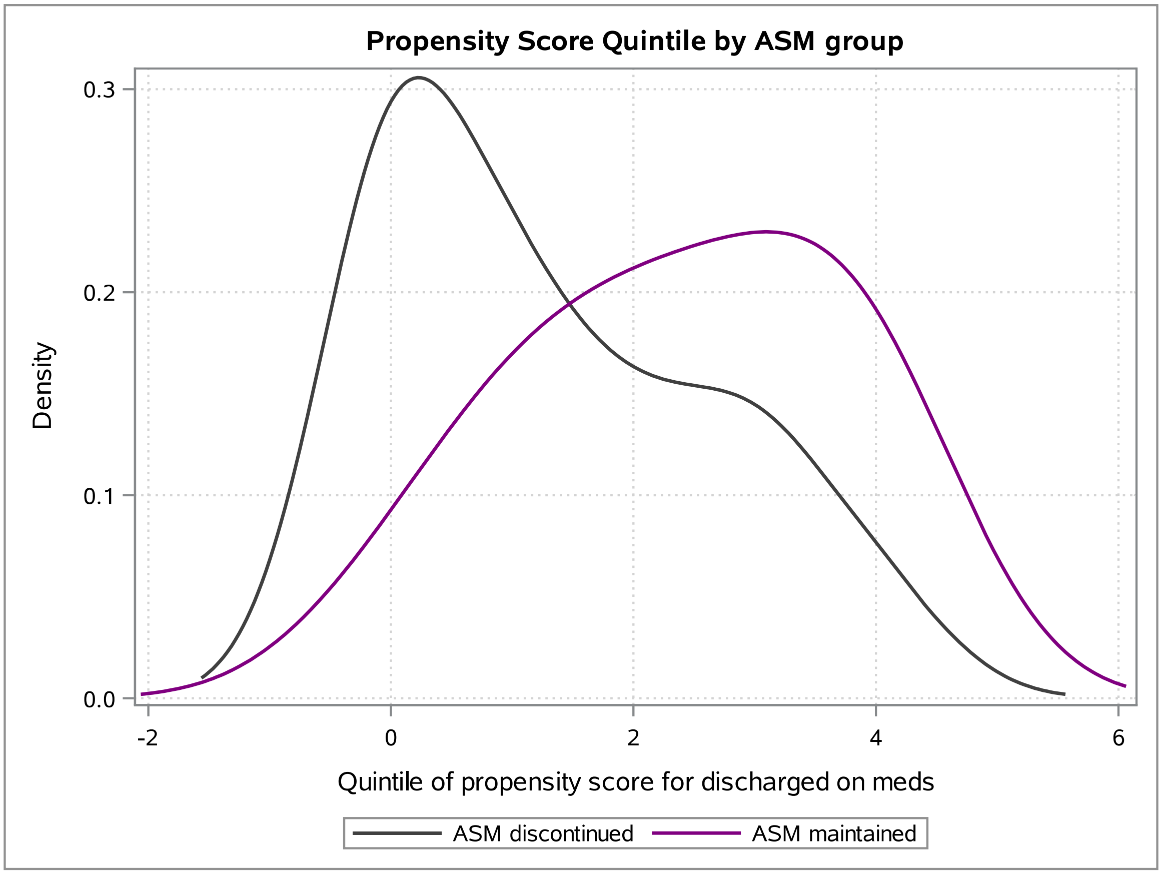

© 2021 Glass HC et al. *JAMA Neurology*.
